# Prevalence of resistance and HIV-1 protease mutation patterns after failures with fosamprenavir-containing regimens

**DOI:** 10.1186/1758-2652-13-S4-P136

**Published:** 2010-11-08

**Authors:** AG Marcelin, C Charpentier, C Ferreira, M Wirden, C Lemarchand, D Descamps, V Calvez

**Affiliations:** 1 Pitie-Salpetriere Hospital, Paris, France; 2 Bichat-Claude Bernard Hospital, Paris, France; 3 ViiV Healthcare, Paris, France; 4 Pitie-Salpetriere Hospital, 83 bd de l'hopital, Paris, France

## Purpose of the study

Fos-Amprenavir is one of the protease inhibitor that is recommended to treat protease inhibitor naïve of experienced patients. Amprenavir and darunavir share at least in part chemical structures and thus a possible selection of close protease gene mutations. Little is known about the frequency and the type of resistance mutations selected after virological failure to Fos-Amprenavir containing regimen. The aim of this study was to determine the genetic patterns and the prevalence of resistance mutations associated to virological failure to Fos-Amprenavir containing regimens in a cohort of naïve and experienced protease inhibitor patients.

## Methods

172 PI experienced patients, treated by r/Fos-APV (100/700 mg BID) and 96 PI naïve patients treated by r/Fos-APV (100/700 mg BID n = 33 and 100/1400 mg OD n = 63) were analyzed. Reverse transcriptase (RT) and protease gene were sequenced and aminoacid changes analyzed before Fos-APV treatment and at time of virological failure. Mutations were analyzed with the ANRS algorithm V18.

## Summary of results

In PI experienced patients, there is a direct link between the number of PI resistance that were present at baseline and the probability of selection of resistance mutation at failure (p=0.01). The most common mutations selected were: V32I, L33F, M46L, I50V, I54L/V, I84V and L90M. In all cases when the protease gene at baseline harbored at least one PI resistance mutation, at least one resistance mutation was added at time of virological failure. Taking into account of the ANRS algorithm, 15% of patient developed a resistance to DRV. In PI naïve patients, only 6% of patients harbored a PI resistance mutation selected at time of virological failure. The most frequent selected mutations were V32I, I47V, I50V and I84V. There is a link between the duration of virological failure under r/Fos-APV and the rate of selected mutation (1% of cases at month 1, 3% at month 3 and 6% at month 6. Taking into account of the ANRS algorithm, only one patient developed a resistance to DRV.

## Conclusions

This study confirms that resistance mutations selected by Fos-APV occurs mainly in PI experienced patients. Except for patients with more than 6 months replication with Fos-APV, the risk to select cross resistance to DRV is very low.

